# Nephroprotective Effects of Wedelolactone against Snake Venom-Induced Acute Kidney Injury: Insights into Experimental Envenomation

**DOI:** 10.1021/acsomega.5c09974

**Published:** 2026-03-04

**Authors:** Mayara A. Romanelli, Pâmella D. Nogueira-Souza, Dayene S. Gomes, Gabriel A. Bastos, Helen M. C. Pinto, Ellen S. Brito, Lucas Albernaz, Tamires Pereira, Janaína Oliveira, Simone S. C. Oliveira, Carolinne S. Amorim, André L. S. Santos, João A. Moraes, Sabrina R. Gonsalez, Paulo A. Melo, Lucienne S. Lara

**Affiliations:** † Instituto de Ciências Biomédicas, 28125 Universidade Federal do Rio de Janeiro, Rio de Janeiro 21941-590, Brazil; ‡ Centro de Pesquisa em Medicina de Precisão, Universidade Federal do Rio de Janeiro, Rio de Janeiro 21941-902, Brazil; § Instituto de Ciências Médicas, Universidade Federal do Rio de Janeiro, Macaé, Rio de Janeiro 27930-560, Brazil; ∥ Instituto de Microbiologia Paulo de Góes, Universidade Federal do Rio de Janeiro, Rio de Janeiro 21941-901, Brazil

## Abstract

Snakebite-induced acute kidney injury (sAKI) is a severe clinical complication associated with *Bothrops* envenomation that can lead to kidney failure. Alternative therapies are needed due to the limitations of antivenom, including variable efficacy, risk of adverse reactions, and significant barriers to access, such as long distances, transportation difficulties, and high costs. This study evaluates the nephroprotective effects of wedelolactone (WEL), a bioactive coumestan derived from *Eclipta prostrata*, known for its anti-inflammatory and antioxidant properties, in a preclinical model of sAKI. Wistar rats were intramuscularly administered 3.5 mg/kg *Bothrops jararacussu* (Bj) venom to induce sAKI, followed by treatment with WEL (2, 5, or 10 mg/kg) 2 h postenvenomation. At 5 mg/kg, WEL effectively mitigated kidney dysfunction, preserving the glomerular filtration rate and reducing proteinuria. Histological analysis revealed a preserved kidney cytoarchitecture and reduced collagen deposition. Biochemical assays indicated that WEL reduced matrix metalloproteinase activity and nitrite levels, key mediators of Bj-induced nephrotoxicity, suggesting a protective role via antioxidant mechanisms. However, WEL at 2 or 5 mg/kg did not prevent Bj-induced muscle damage. Additionally, the 10 mg/kg dose of WEL administered to Bj rats was associated with kidney injury, limiting its potential for clinical application. Notably, WEL did not impair kidney function in healthy rats. These findings underscore the translational potential of WEL as a complementary therapeutic approach for managing sAKI and reducing the long-term burden of snakebite-related kidney disease.

## Graphical abstract

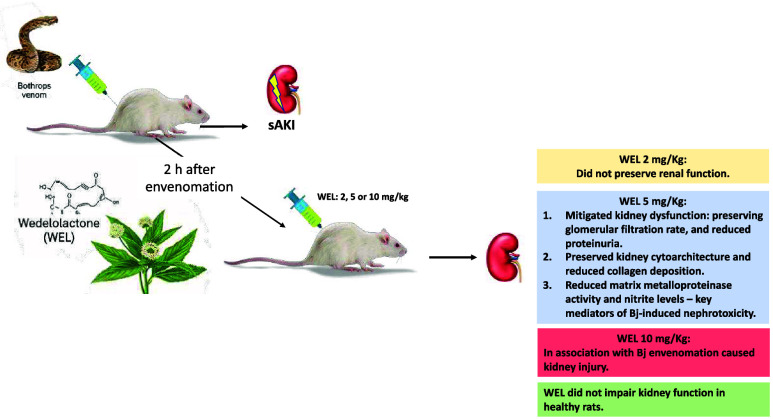

## Introduction

Snakebites represent a significant public health concern and are classified as a neglected tropical disease by the World Health Organization. They predominantly affect impoverished populations living in rural areas.^1–4^ In Latin America, most snakebites are caused by *Bothrops* snakes, leading to complications such as hemorrhage, edema, and necrosis.^5–7^ While hemorrhage is often the primary cause of death, snakebite-induced acute kidney injury (sAKI) is another potentially fatal clinical complication that can progress to chronic kidney failure.^8^

Timely administration of antivenom therapy, along with appropriate supportive care, is essential to reduce morbidity and mortality associated with sAKI. However, antivenom treatment has several limitations: (i) Conventional antivenom exhibits limited efficacy against local tissue damage and presynaptic neurotoxicity, especially when administration is delayed;^9^ (ii) Only a small fraction of the IgG present is specific to the venom, requiring higher doses to achieve therapeutic effects;^9^ (iii) Antivenom therapy may lead to early adverse reactions, occurring within hours of infusion and manifesting as urticaria, itching, bronchospasm, angioedema, colic, nausea, and hypotension;^10^ (iv) Barriers to access, including long distances, lack of transportation, inadequate storage infrastructure, antivenom shortages, and high cost that hinder timely treatment.^11^ These limitations heighten the risk of sAKI and its long-term consequences.^12,13^ In the absence of effective pharmacological therapies, dialysis remains the only available intervention. These challenges underscore the urgent need for novel complementary strategies to improve snakebite management and prevent sAKI.

Depending on fang size, venom is injected either subcutaneously or intramuscularly.^9^ In this context, we previously developed a preclinical model of sAKI by intramuscular (IM) administration of 3.5 mg/kg *Bothrops jararacussu* (Bj) venom to Wistar rats, simulating clinical scenarios. This model is characterized by early glomerular hyperfiltration, proteinuria, albuminuria, and decreased fractional sodium excretion (FE_Na_) that developed 24 h after Bj administration, along with severe kidney tubular injury and muscle damage. At 72 h postvenom administration, there is a decrease in glomerular filtration rate (GFR) and urine output.^14^

*Eclipta prostrata* (EP), a medicinal plant widely used in Asia and South America, has a long history of traditional application as a topical antivenom for snakebites.^15^ Extracts of EP and its major coumestan constituent, wedelolactone (WEL), have demonstrated inhibitory activity against snake venoms from multiple genera, including *Crotalus durissus terrificus*, *Bothrops jararaca*, *Lachesis muta*, and *Agkistrodon*, particularly by reducing hemorrhagic, myotoxic, and enzymatic effects.^16–21^ Importantly, these antivenom properties were reported primarily under *in vitro* preincubation conditions, in which WEL or EP extracts were mixed with venom before administration, an approach that does not replicate clinical reality.

Beyond its antivenom effects, WEL exhibits a broad pharmacological profile, including antiviral, antihypertensive, antihepatotoxic, immunomodulatory, anti-inflammatory, and antitumor activities.^22–26^ In kidney research, WEL has been shown to suppress inflammation and apoptosis in sepsis-induced kidney injury by upregulating PTPN2 in HK-2 cells.^27^ Additionally, WEL alters cisplatin pharmacokinetics, reducing its renal accumulation and attenuating cisplatin-induced acute kidney injury in mice.^28^

Despite these findings, to our knowledge, no study to date has evaluated whether WEL can protect the kidney from injury induced by snake venom *in vivo* under conditions that reflect postenvenomation treatment. Moreover, although many natural antioxidants and anti-inflammatory compounds have been investigated in experimental envenomation models, WEL is mechanistically distinct: unlike general antioxidant molecules, WEL exhibits a unique combination of broad anti-inflammatory actions, potential modulation of protein tyrosine phosphatases (e.g., PTPN2), and previously demonstrated interactions with snake venom components. Thus, this study aims to determine whether these properties translate into nephroprotection during sAKI induced by Bj venom administered intramuscularly. By investigating WEL as a postvenom therapeutic, rather than as a preincubated inhibitor, this study advances the field beyond existing evidence on EP extracts and WEL’s known pharmacological effects, offering new insight into its translational potential as an adjunctive strategy for snakebite management.

## Results

### WEL (5 mg/kg) Prevented Bj-Induced Kidney Function Impairment, but Not Muscle Lesion

Intramuscular administration of Bj caused muscle injury when compared to the control (Ctrl), with a nearly 14-fold increase (Figure 1). Treatment with WEL did not prevent muscle damage, and the higher WEL dose (10 mg/kg) exacerbated Bj-induced muscle injury.

**Figure 1:**
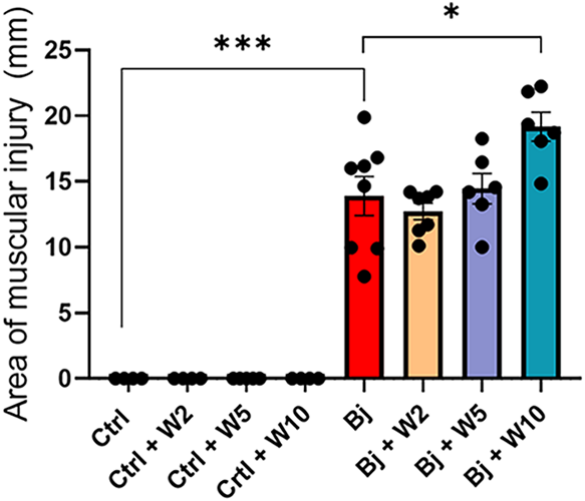
WEL treatment did not prevent Bj-induced muscle injury. Quantification of the muscular injury after 24 h of the intramuscular administration of Bj with or without WEL treatment. The results are expressed as mean ± SEM. *p < 0.05, ***p < 0.001. Ctrl: control; Bj: *B. jararacussu*; W: Wedelolactone.

Physiological and kidney filtration function parameters are described in Table 1. Water intake (WI) remained unchanged under all conditions. The preclinical model of sAKI, as described in ref 14, was successfully reproduced. After 24 h, Bj-treated rats exhibited the following: (i) 36% increase in 24 h urinary volume (UV); (ii) a 2-fold increase in glomerular filtration rate (GFR), accompanied by a marked accumulation of plasma creatinine concentration (PCre) (100%) and blood urea nitrogen (BUN) (54%); (iii) intense proteinuria and albuminuria, with a 2-fold increase; and (iv) an 80% elevation in the proteinuria/UCre (Ptn/UCre) ratio.

**Table 1:** Impact of WEL Treatment on Physiological Parameters and Kidney Function of Ctrl and Bj Rats^c^

	Ctrl (*n* = 12)	Ctrl + W2 (*n* = 12)	Ctrl + W5 (*n* = 12)	Ctrl + WlO (*n* = 12)	Bj (*n* = 12)	Bj + W2 (*n* = 12)	Bj + W5 (*n* = 12)	Bj + WlO (*n* = 12)
Physiological Parameter								
UV	11 ± 0.7	14 ± 0.8	12 ± 1.0	12 ± 0.9	15 ± 1.2^a^	13 ± 1.1	11 ± 0.8^b^	14 ± 0.7
WI	33 ± 3.4	35 ± 3.7	29 ± 2.3	28 ± 3.0	33 ± 3.2	33 ± 2.1	30 ± 2.9	27 ± 1.2
Renal Filtration Function								
UCre	41 ± 4.2	32 ± 3.7	45 ± 1.2	41 ± 6.0	34 ± 2.4	31 ± 2.7	38 ± 4.4	38 ± 3.2
PCre	0.3 ± 0.04	0.2 ± 0.04	0.2 ± 0.04	0.6 ± 0.12^a^	0.6 ± 0.05^a^	0.5 ± 0.05	0.3 ± 0.05^b^	0.3 ± 0.06^b^
GFR	1165 ± 107	1874 ± 680	1301 ± 652	906 ± 264	2285 ± 425^a^	601 ± 80^b^	1312 ± 230^b^	1305 ± 205^b^
Ptn/UCre	0.4 ± 0.04	0.6 ± 0.07	0.5 ± 0.16	0.5 ± 0.06	0.8 ± 0.08^a^	0.7 ± 0.05	0.5 ± 0.04^a^	0.7 ± 0.10
BUN	41 ± 4.2	47 ± 3.9	43 ± 8.9	43 ± 4.5	63 ± 3.5^a^	48 ± 4.9	43 ± 2.4^b^	52 ± 4.4
Proteinuria	20 ± 1.6	20 ± 1.0	18 ± 3.0	27 ± 3.2	43 ± 6.6^a^	26 ± 2.4^b^	22 ± 3.0^b^	26 ± 4.2^b^
Albuminuria	2.1 ± 0.1				3.8 ± 0.6^a^	3.8 ± 0.5	5.2 ± 0.4	3.7 ± 0.1

a *p* < 0.05 compared with Ctrl.

b *p* < 0.05 compared with Bj.

c Abbreviations: UV: urinary volume in mL/24 h; WI: water intake in mL/24 h; UCre: urinary creatinine in mg/dL; PCre: plasmatic creatinine in mg/dL; GFR: glomerular filtration rate in mL/min; Ptn: proteinuria in in mg/dL; BUN: blood urea nitrogen in in mg/dL. Albuminuria was measured as arbitrary units. The data are presented as mean ± SEM.

The dose–response effect of WEL on urine volume, BUN, and the Ptn/UCre ratio appeared to be biphasic. A dose of 5 mg/kg WEL effectively prevented the increase in these parameters, whereas doses of 2 and 10 mg/kg resulted in values similar to those observed in the Bj. WEL also prevented the increase in GFR and proteinuria at 2 mg/kg and PCre at 5 mg/kg. Albuminuria remained unchanged across all WEL doses.

### WEL Treatment Prevented the Bj-Impaired Kidney Na^+^ Handling, but Did Not Affect Kidney Cortical Primary Na^+^ Transporter Activities

As expected, Bj-induced hyperfiltration resulted in a 95% increase in sodium filtered load (FLNa). However, Bj-treated rats exhibited a 40% reduction in urinary sodium concentration (U_Na_V), accompanied by a 45% decrease in FE_Na_. Treatment with the lowest WEL dose (2 mg/kg) effectively prevented the Bj-induced impairment in Na^+^ handling. Notably, WEL treatment did not affect Na^+^ handling in Ctrl (Table 2).

**Table 2:** WEL Rescued Bj-Impaired Kidney Na^+^ Handling^c^

	Ctrl (*n* = 12)	Ctrl + W2 (*n* = 12)	Ctrl + W5 (*n* = 12)	Ctrl + W10 (*n* = 12)	Bj (*n* = 12)	BJ + W2 (*n* = 12)	BJ + W5 (*n* = 12)	Bj + W10 (*n* = 12)
FL_Na_	215 ± 19	329 ± 129	473 ± 145	156 ± 80	420 ± 77^a^	153 ± 47^b^	306 ± 47	254 ± 42
U_Na_V	2.5 ± 0.2	1.8 ± 0.3	1.8 ± 0.2	2.5 ± 0.2	1.5 ± 0.2^a^	2.5 ± 0.1^b^	1.7 ± 0.2	1.4 ± 0.1
FE_Na_	1.1 ± 0.2	0.4 ± 0.06	0.4 ± 0.06	1.2 ± 0.2	0.6 ± 0.06^a^	1.4 ± 0.3^b^	0.6 ± 0.1	0.5 ± 0.07

a *p* < 0.05 compared with CTRL.

b *p* < 0.05 compared with Bj.

c Abbreviations: FL_Na_: filtered load Na^+^ in mEq/min; U_Na_V: urinary Na^+^ concentration in mEq/100 g of body weight; FE_Na_: fractional excretion of Na^+^, the percentage of FL_Na_ that was excreted in the urine. The data are presented as mean ± SEM.

The protein content of (Na^+^+K^+^)-ATPase remained unchanged across all experimental groups (Figure 2A). Bj treatment induced a 27% increase in (Na^+^+K^+^)-ATPase activity (in nmol Pi·mg^–1^·min^–1^: 393 ± 16 in Ctrl vs 499 ± 32 in the Bj; Figure 2B) and an increase in 82% of Na^+^-ATPase activity (in nmol Pi·mg^–1^·min^–1^: 38 ± 6 in the Ctrl vs 69 ± 4 in the Bj; Figure 2C). Treatment with 5 mg/kg WEL failed to prevent the Bj-induced enhancement in Na^+^ transport. Additionally, WEL administration in the Ctrl had no effect on enzyme activities.

**Figure 2:**
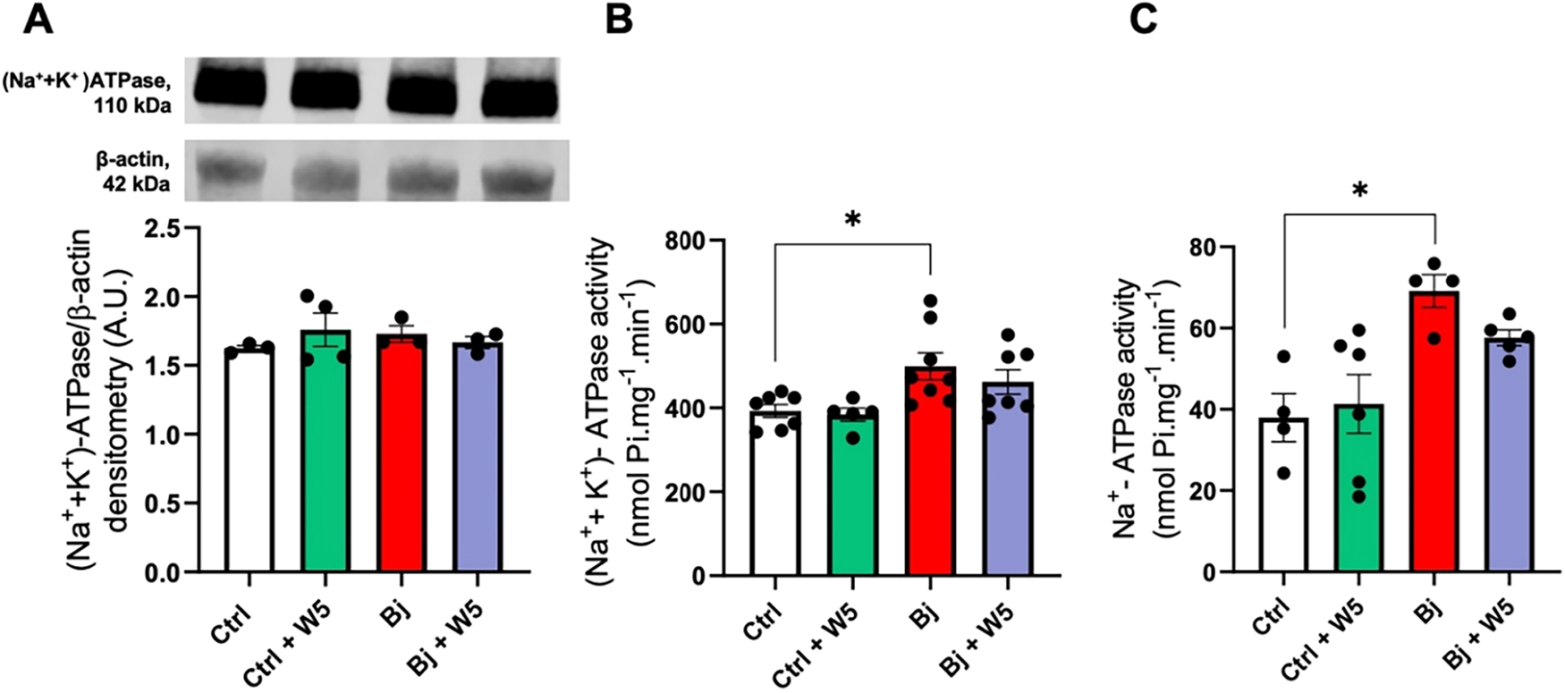
Bj augmented kidney cortical primary Na^+^ transporter activities, but WEL (5 mg/kg) treatment did not prevent it. (A) (Na^+^ + K^+^)-ATPase protein content. Upper panel: representative images of (Na^+^ + K^+^)-ATPase and β-actin detections. Lower panel: densitometric analysis of the immunoreactive band ratio between (Na^+^ + K^+^)-ATPase and β-actin detections. (B) Ouabain-sensitive (Na^+^ + K^+^)-ATPase activity. (C) Ouabain-resistant, furosemide-sensitive Na^+^-ATPase activity. The results are expressed as mean ± SEM. *p < 0.05. Ctrl: control; Bj: *B. jararacussu*; W: Wedelolactone.

### WEL (5 mg/kg) Prevented Bj-Induced Nephrotoxicity

Histological analysis of the kidneys was performed using hematoxylin-eosin (HE) staining (Figure 3). Ctrl kidneys displayed normal cytoarchitecture with clearly proximal and distal tubules. The glomeruli exhibited a typical appearance (Figure 3A,B). In contrast, Bj caused cortical injury characterized by loss of the brush border (arrow), degeneration of luminal tubular cells (open rectangle), glomerular segmentation (asterisk), and an increase in Bowman’s space (black square) (Figure 3C,D).

**Figure 3:**
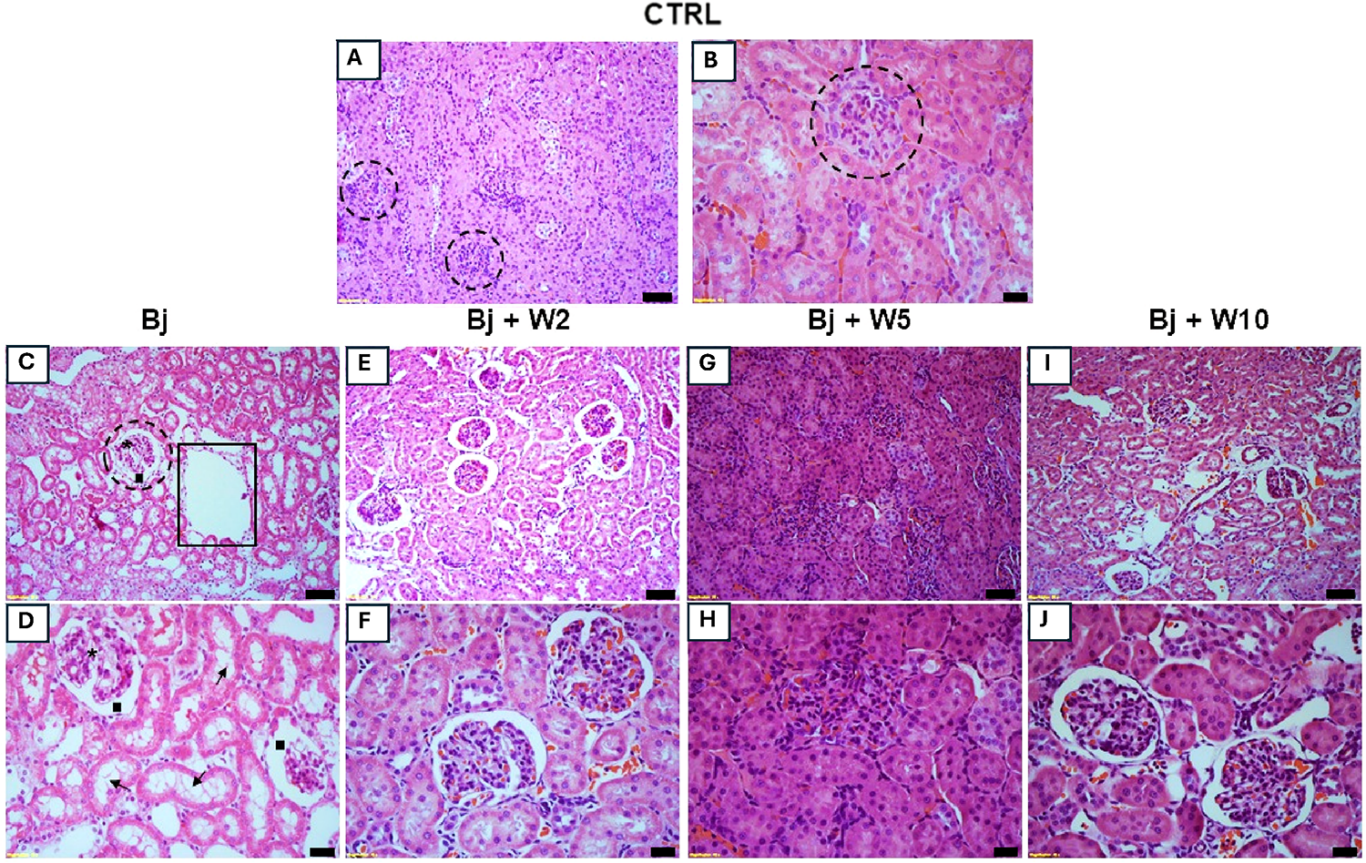
WEL (5 mg/kg) treatment blocked Bj-induced kidney damage. Representative photomicrographs of cortical kidney sections (3 μm) stained with HE. Kidney damage is demonstrated as loss of the brush border (arrow), degeneration of luminal tubular cells (open rectangle), glomerular segmentation (asterisk), and increased Bowman’s space (black square). (A and B) Ctrl, (C and D) Bj, (E and F) Bj + W2, (G and H) Bj + W5, (I and J) Bj + W10. For each group, *n* = 4. Ctrl: control; Bj: *B. jararacussu*. Scale bars represent: (A), (C), (E), (G), and (I): 50 μm; (B), (D), (F), (H), and (J): 20 μm.

Histomorphometry evaluations revealed that the Bj resulted in a 30% increase in renal corpuscle size (Figure 4A) and a 97% increase in the Bowman’s space (Figure 4C). However, Bj did not affect the glomerular area (Figure 4B). WEL (5 mg/kg) preserved kidney cytoarchitecture, as shown by comparing Figure 3F,I with Figure 3B,D (Bj-treated rats). At the same dose, WEL inhibited the expansion of the renal corpuscle and Bowman’s space (Figure 4A,4C, respectively). However, neither 2 nor 10 mg/kg WEL prevented morphological changes and histomorphometry alterations observed in Bj (Figure 3E–J).

**Figure 4:**
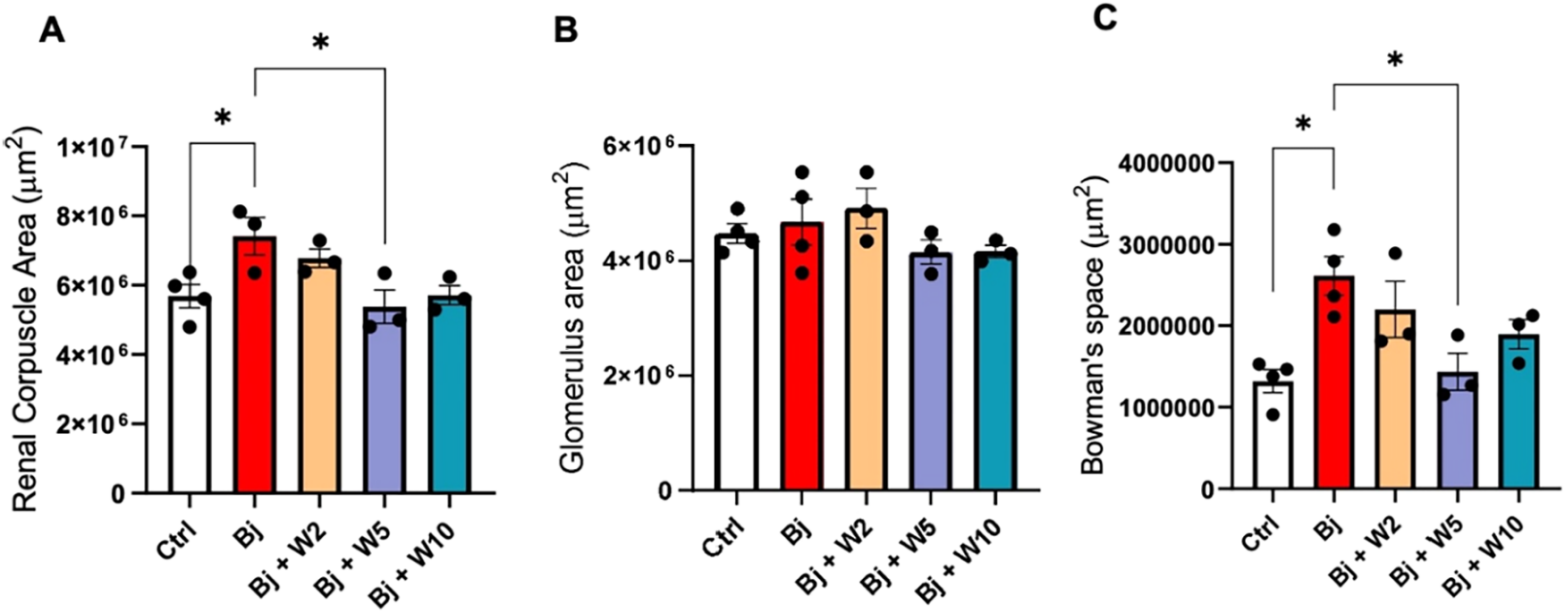
WEL treatment preserved glomerular histomorphometric parameters in Bj rats. Histomorphometric analyses from the images obtained in Figures 3 and 4 at 400× magnification. (A) Renal corpuscle area; (B) glomerular area; (C) Bowman’s space. The results are expressed as mean ± SEM. *p < 0.05. Ctrl: control; Bj: *B. jararacussu*; W: Wedelolactone.

Bj rats exhibited collagen accumulation around cortical and medullary tubules (Figures 6A and 7A, compared with Figure 5). Quantification of collagen in kidney tissue revealed that collagen deposition was more pronounced in the medulla than in the cortex, accounting for 60% and 40% of total collagen deposition, respectively (Figures 6E and 7E, compared with Figure 5). Treatment with WEL (5 mg/kg) blocked collagen deposition in both the kidney cortex and medulla, whereas doses of 2 and 10 mg/kg did not (Figures 6 and 7, compared with Figure 5).

**Figure 5:**
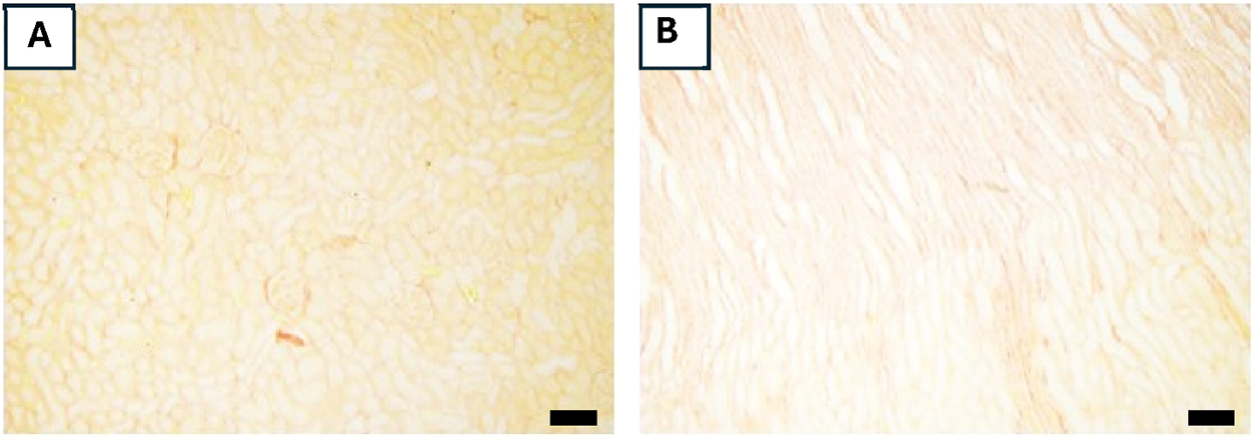
Collagen accumulation in the kidney cortical and medullary parenchyma in control rats. Representative photomicrographs (100× magnification) of cortical (A) and medullary (B) kidney sections (3 μm) stained with picrosirius red. Scale bars represent: (A) and (B): 50 μm.

**Figure 6:**
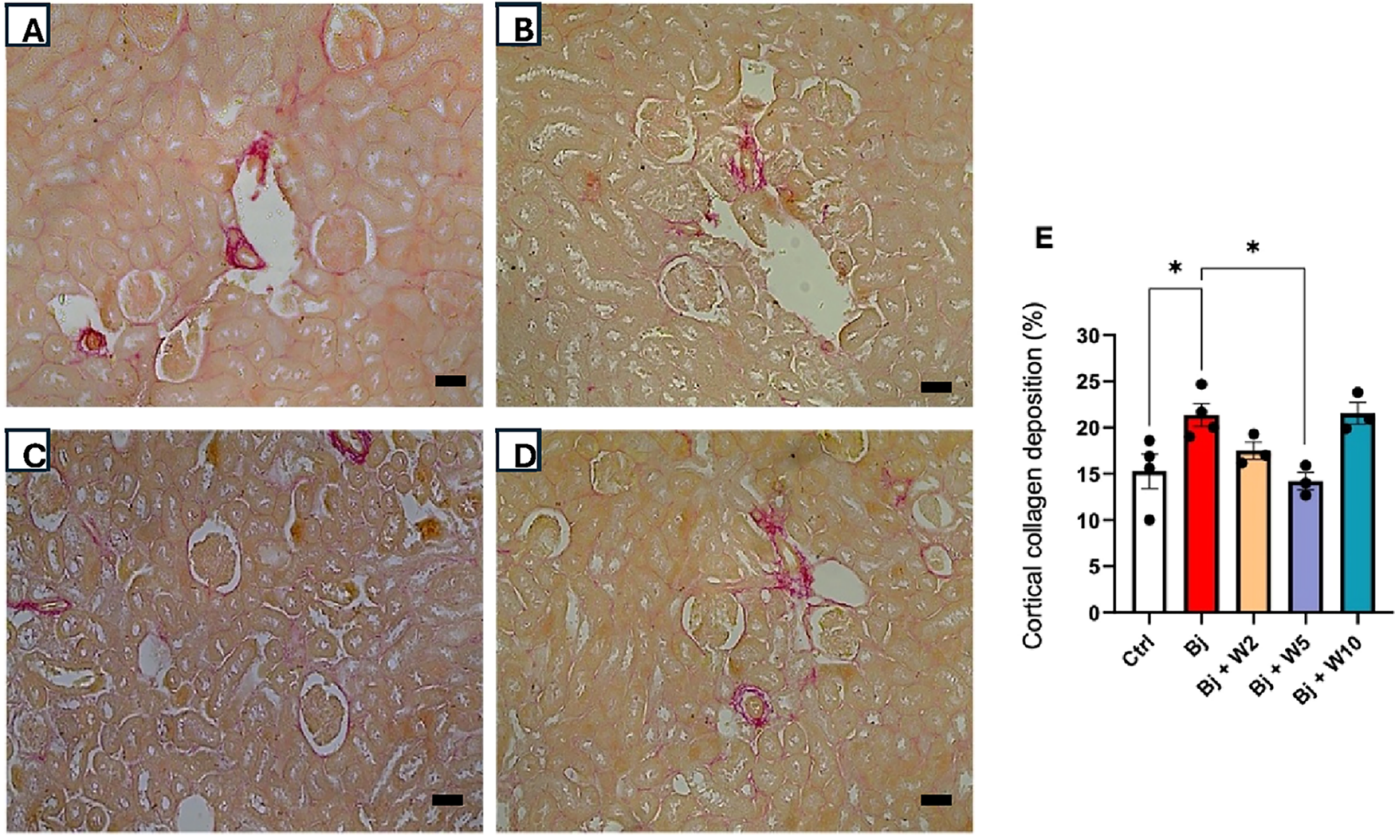
WEL treatment impeded collagen accumulation in the kidney cortical parenchyma induced by Bj. Representative photomicrographs (200× magnification) of cortical kidney sections (3 μm) stained with picrosirius red. (A) Bj, (B) Bj + W2, (C) Bj + W5, (D) Bj + W10, and (E) quantification of collagen deposition in the kidney cortex. The results are expressed as mean ± SEM. For each group *n* = 4. *p < 0.05. Ctrl: control; Bj: *B. jararacussu*; W: Wedelolactone. Scale bars represent: (A), (B), (C), and (D): 50 μm.

**Figure 7:**
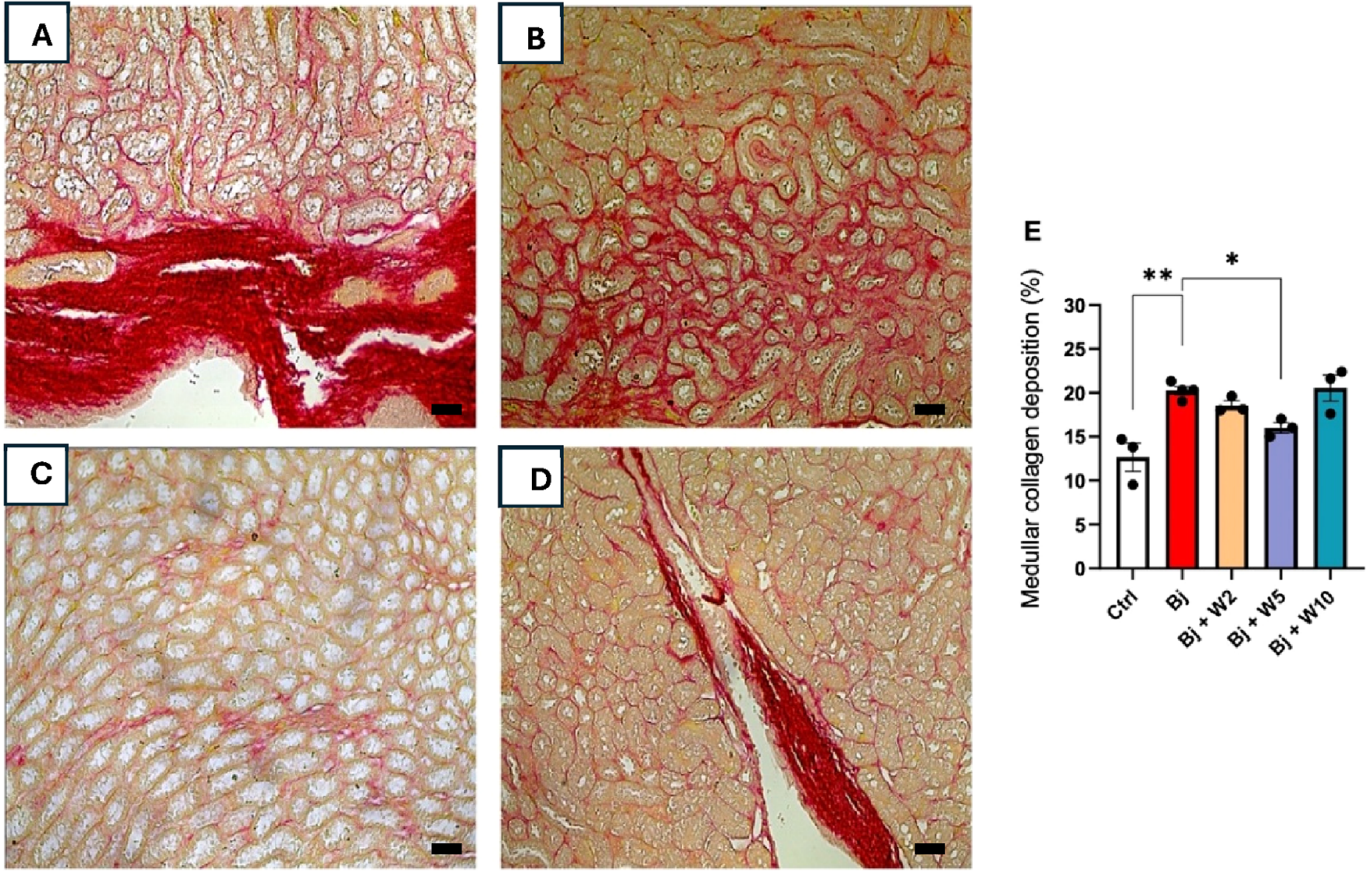
WEL treatment impeded collagen accumulation in the kidney medullary parenchyma induced by Bj. Representative photomicrographs (200× magnification) of cortical kidney sections (3 μm) stained with picrosirius red. (A) Bj, (B) Bj + W2, (C) Bj + W5, (D) Bj + W10, and (E) quantification of collagen deposition in the kidney medulla. The results are expressed as mean ± SEM. For each group *n* = 4. *p < 0.05. Ctrl: control; Bj: *B. jararacussu*; W: Wedelolactone. Scale bars represent: (A), (B), (C), and (D): 50 μm.

### Mechanism of Action of WEL: Blocking MMP Activity and Oxidative Stress Induced by Bj

Bj rats exhibited a 20% increase in total matrix metalloproteinase (MMP) activity compared to that of Ctrl rats (Figure 8A,8B). Treatment with WEL (5 mg/kg) normalized total MMP activity in the kidney cortex. The involvement of MMPs was confirmed by the addition of 1,10-phenanthroline (PHEN), a classical nonspecific MMP inhibitor, to kidney cortex homogenates (Figure 7C). WEL (5 mg/kg) did not alter the MMP activity in the Ctrl (Figures 8A,8B).

**Figure 8:**
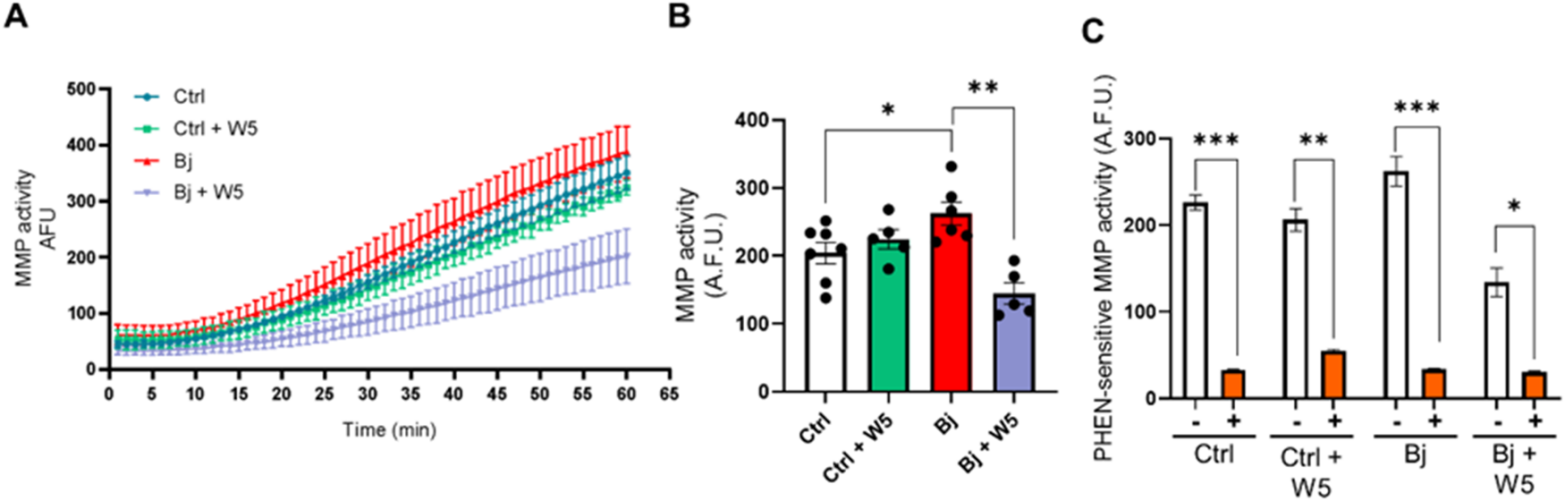
Treatment with 5 mg/kg WEL blocked kidney cortical MMP activity induced by Bj. (A) Kinetics of the MMP activity. (B) End-point MMP activity was measured at 60 min. (C) 1,10-phenanthroline (PHEN)-sensitive MMP activity. The proteolytic activity of the fluorescent substrate was measured in the absence (−) or presence (+) of 5 mM PHEN, a pan metalloproteinase inhibitor. Values are expressed as arbitrary fluorescent units (A.F.U.). The data are presented as the mean ± SEM. *p < 0.05; ** *p* < 0.01; ***p < 0.0001. Ctrl: control; Bj: *B. jararacussu*; W: Wedelolactone.

We investigated key components of endoplasmic reticulum stress (ER stress), including GRP78, CHOP, ATF4, Bcl-2, and Caspase 12, in the kidney cortex (Figure 9). The immunoblotting assays revealed no significant differences between the Bj and Ctrl or among the treated groups. Given the antioxidant properties of WEL,^26,27^ we measured nitrite concentration (Figure 10A) and myeloperoxidase (MPO) activity (Figure 10B) in the kidney cortex as markers of oxidative stress. Bj induced a 25% increase in the nitrite concentration (Figure 10A) and a 22% increase in MPO activity (Figure 10B). However, WEL treatment only restored the nitrite concentration. Ctrl treated with WEL showed no changes in these molecular markers.

**Figure 9:**
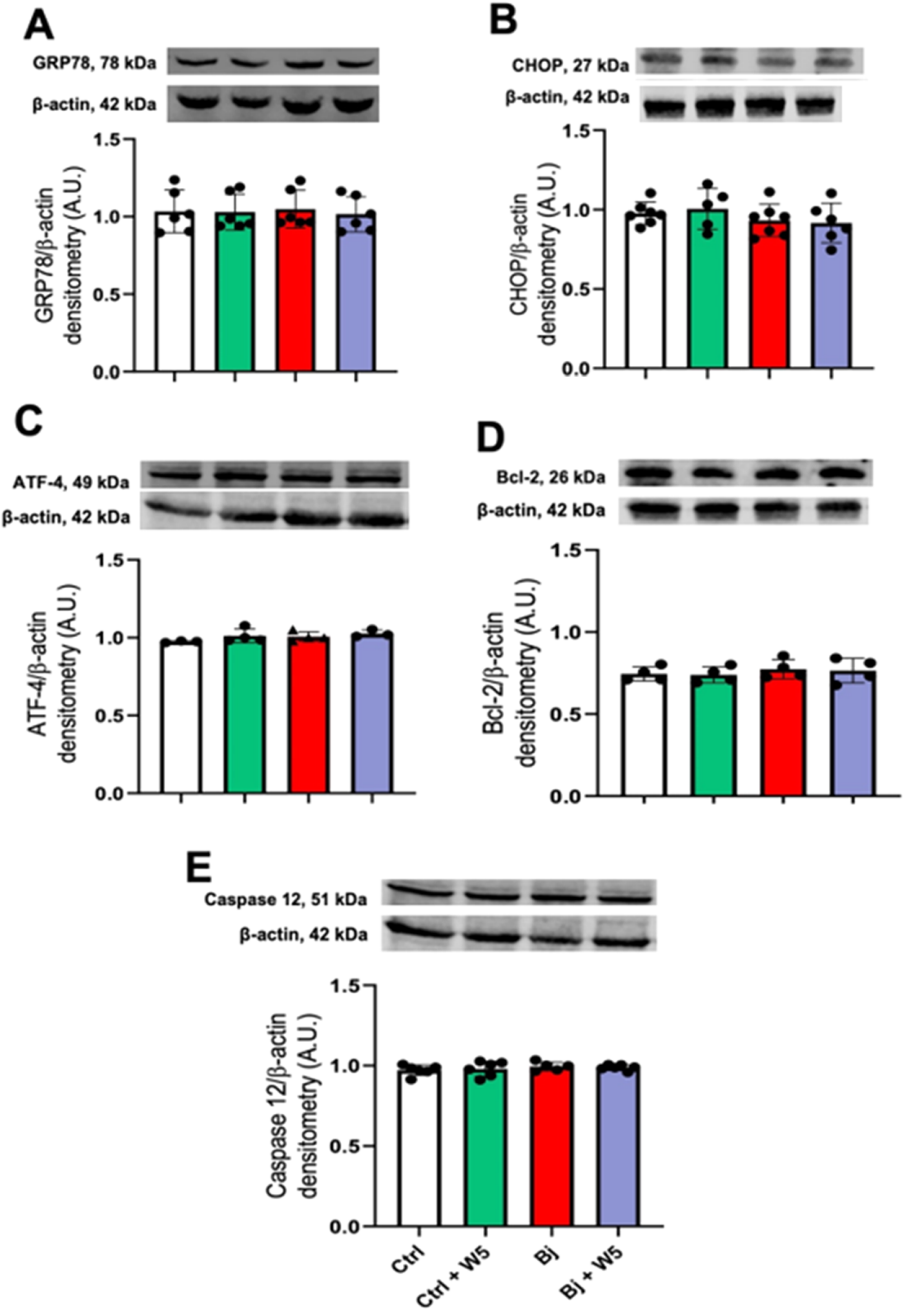
ER-stress markers remained unchanged following both Bj envenomation and WEL treatment. (A) GRP78; (B) CHOP; (C) ATF4; (D) Bcl-2; and (E) Caspase 12. Upper panels: representative image of the immunoblots; lower panels: densitometric measurement of the immunobands. β-actin was used as the loading control. Values are expressed as arbitrary units (A.U.). The data are presented as the mean ± SEM. Ctrl: control; Bj: *B. jararacussu*; W: Wedelolactone.

**Figure 10:**
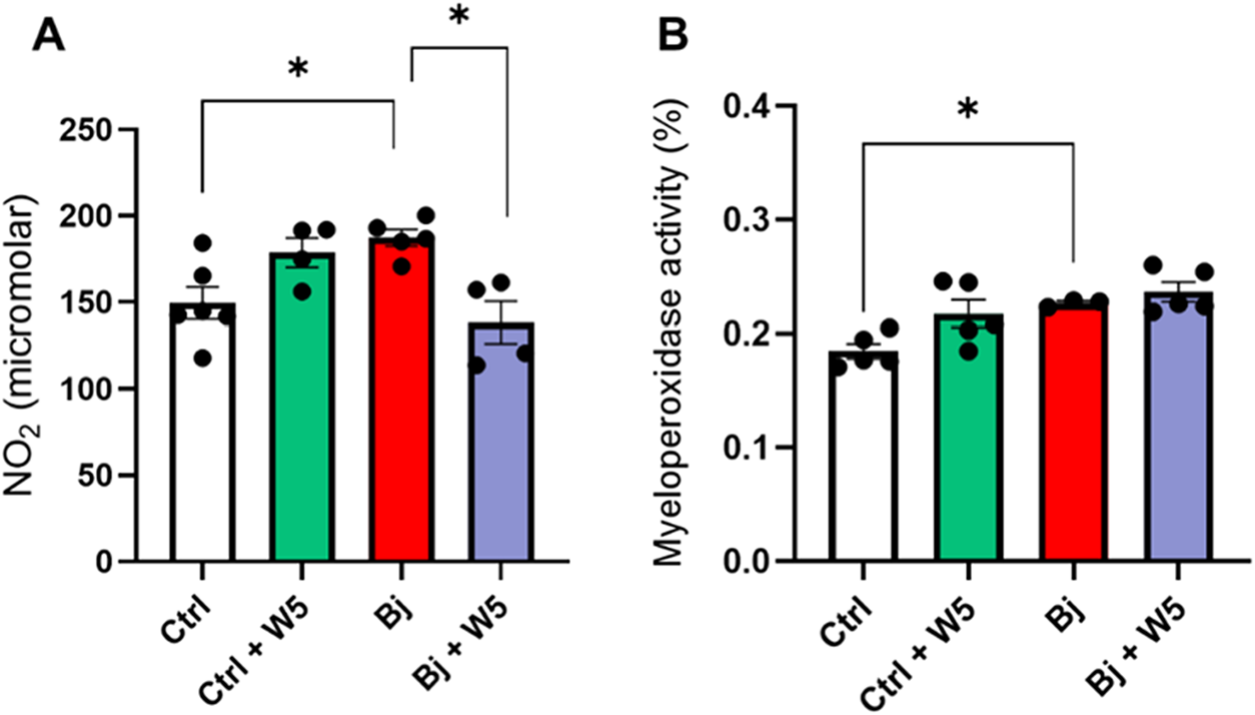
WEL treatment partially reduced stress oxidative markers in the kidney cortex induced by Bj. (A) The concentration of nitrite in the kidney cortical homogenate. (B) Myeloperoxidase activity was measured in the cortical region of kidney samples. The data are presented as the mean ± SEM. *p < 0.05. Ctrl: control; Bj: *B. jararacussu*; W: Wedelolactone.

## Discussion

This study provides clear evidence that WEL exerts a nephroprotective effect against early functional and structural kidney damage induced by Bj. Importantly, WEL demonstrated no signs of nephrotoxicity when administered to healthy rats. The observed protective effects are likely mediated by the attenuation of oxidative stress in the renal cortex and the inhibition of MMP activation, both of which are critical contributors to Bj-induced kidney injury. These findings, in conjunction with other studies,^25,26,28,29^ position WEL as a promising complementary therapeutic candidate for mitigating sAKI.

Available pharmacokinetic data indicate that wedelolactone (WEL) exhibits rapid absorption, moderate oral bioavailability, and wide tissue distribution in rodents, including detectable levels in the liver, kidneys, and even the brain. WEL undergoes rapid hepatic metabolism through hydrolysis, lactone ring opening, methylation/demethylation, and glucuronidation, with urinary excretion of its metabolites.^30^ Across several animal studies, WEL has been well tolerated at therapeutic doses, with toxicity largely restricted to *in vitro* cancer-specific models, although human safety data remain limited.^31^ These pharmacokinetic features support the rationale for testing low doses such as 2, 5, and 10 mg/kg *in vivo*. Furthermore, the chosen doses for this study were previously used in studies that determined the *in vivo* WEL’s antimyotoxic activity of different snake venoms.^16,32,33^

We used a preclinical sAKI model to test new pharmacological substances aimed at preventing clinical sAKI and mitigating its outcome.^14^ The correlation between kidney cytoarchitectural changes and AKI symptoms is well established, particularly with glomerular and tubulointerstitial alterations.^34^ Glomerular damage is linked to hyperfiltration,^35^ and the extent of structural changes correlates with proteinuria. These outcomes reflect disruptions in the basement membrane, mesangial matrix, and podocyte cytoskeletal proteins, compromising the filtration barrier. These effects are likely due to direct venom action on renal tissue.^36^

We evaluated three different doses of WEL (2, 5, and 10 mg/kg), observing a biphasic dose–response effect on urine volume, BUN, ptn/UCre ratio, and collagen accumulation. WEL (2 mg/kg) partially prevented sAKI by blocking the augmented GFR and proteinuria but not the alterations of other renal parameters, in part due to the rapid metabolism. Although 5 mg/kg provides nephroprotection, 10 mg/kg WEL showed injury profiles similar to those of Bj alone. Specifically, in the Bj rats treated with 10 mg/kg WEL, increased urine volume, BUN, ptn/UCre ratio, albuminuria, and collagen deposition were observed. Since Bj + W10 rats, but not Ctrl + W10, exhibited exacerbated muscle injury, we suggest that WEL at a higher dose, when administered in the context of Bj envenomation, may induce renal damage. We can speculate that envenomation may induce WEL accumulation in the kidney or induce WEL’s nephrotoxic metabolites. Although previous studies indicate that 0.2% DMSO (the vehicle for WEL) is a safe concentration,^37–39^ the absence of a vehicle-control group prevents us from excluding a potential effect of DMSO on the kidney.

The focus of our mechanistic investigation was intentionally placed on the most effective dose to elucidate the pathways responsible for WEL’s protective action. Thus, we selected 5 mg/kg WEL to explore its molecular mechanisms, focusing on the MMP activity and oxidative stress. This dose was also the chosen dose administrated *in vivo* models of myotoxic activity induced by snake venom.^33^ The loss of efficacy at higher concentrations may suggest a narrow therapeutic window or potential off-target effects, a phenomenon often observed with high-dose antioxidant polyphenols. We emphasize the need for future studies focused on clinical prospection and therapeutic index determination, which will require broader dose–response evaluations, additional toxicological analyses, and the inclusion of complementary nonrodent *in vivo* models.

Mice *extensor digitorum longus* and *soleus* muscle exposure to Bj for 60 min caused a 10 times increase in creatinine release in comparison to the basal levels. This increase is similar to other crotaline venoms (*Agkistrodon contortrix laticinctus*, *Crotalus viridis viridis*, *Crotalus durissus terrificus*).^40^ Extensive muscle degradation resulting from Bj proteolytic activity leads to significant increases in blood urea nitrogen and creatinine, despite the hyperfiltration observed 24 h postenvenomation. The massive protein release from muscle to the kidneys affects the renal cytoarchitecture and contributes to proteinuria. Despite not preventing myotoxicity, WEL (5 mg/kg) effectively prevented sAKI. Although WEL has been shown to inhibit PLA_2_ activity *in vitro*, this effect may not translate *in vivo* under our experimental conditions. Previous studies demonstrated that preincubation of venom with WEL or EP extract prevented myotoxicity.^21,29,41^ However, in our study, WEL was administered 2 h postenvenomation, by which time PLA_2_ activity had likely initiated irreversible muscle damage. This distinction is critical: WEL did not reverse muscle injury but still conferred clear nephroprotection, suggesting that its renoprotective mechanisms operate independently of early PLA_2_-mediated toxicity. Albuminuria may be a consequence of muscle damage not prevented by WEL nephroprotection.

Impaired tubular Na^+^ handling is a hallmark of kidney dysfunction, leading to elevated blood pressure, proteinuria, glomerular hyperfiltration, and reduced responsiveness to intrarenal blockade of the renin-angiotensin system (RAS) [revised in ref 42]. The primary Na^+^ transporters [(Na^+^ + K^+^)-ATPase and Na^+^-ATPase] are responsible for establishing electrochemical gradients crucial for Na^+^ and water reabsorption and hydroelectrolyte balance.^43^ As Bj increases GFR, it also augments FLNa, accompanied by reductions in UNaV and FENa, partially due to increased cortical (Na^+^ + K^+^)-ATPase and Na^+^-ATPase activities. Similar results were described for *Bothrops alternatus* venom, where increased (Na^+^ + K^+^)-ATPase activity was paradoxically associated with FENa in the first 6 h postenvenomation.^44^ Conversely, studies by Guerrero et al. demonstrated a decrease in FL_Na_ and cortical (Na^+^ + K^+^)-ATPase activity, with no changes in U_Na_V 24 h after envenomation with *Bothrops jararaca* venom.^45^ These discrepancies underscore the need for more comprehensive species-specific studies to clarify *Bothrops* venom effects on renal Na^+^ handling and transporters. Treatment with 2 mg/kg WEL prevented increases in FLNa and reductions in UNaV and FENa. WEL (5 mg/kg) did not reverse the Bj-augmented Na^+^ transport and U_Na_V and FE_Na_. WEL directly inhibits (Na^+^ + K^+^)-ATPase activity, with an IC_50_ of 0.7 μM. Because (Na^+^ + K^+^)-ATPase activity is measured as the difference in ATPase activity in the presence and absence of ouabain, it is plausible that WEL administered *in vivo* interfered with the binding of ouabain to the enzyme, thereby affecting the detection of enzyme activity *in vitro*.

Bj venom contains multiple nephrotoxic components, including snake venom metalloproteinases (SVMPs), snake venom serine proteases (SVSPs), l-amino acid oxidase (LAO), and PLA_2_. Their combined or isolated actions contribute to ROS generation, elevated intracellular Ca^2+^, mitochondrial dysfunction, and inflammatory responses marked by increased TNFα and interleukins, all of which disrupt renal hemodynamics.^46^ We demonstrated that the direct action of Bj leads to increased MMP activity and elevated nitrite tissue concentration, both of which are related to kidney fibrosis.^47^ The second most abundant Bj component is SVMPs,^48^ which are classified as P–I, P–II, and P–III based on their domains. Their metalloproteinase domain mediates ECM degradation and destabilizes cell-matrix interactions in the kidney.^47^ We observed increased renal MMP activity after Bj injection, reversed by WEL. In the kidney, MMP-2 and MMP-9 are the main MMPs involved in kidney fibrosis and the development of chronic kidney diseases, leading us to postulate that WEL may inhibit these MMP isoforms, contributing to less collagen deposition.^49^ Our study demonstrated that WEL treatment caused in the Bj rats both a decrease in MMP activity and collagen deposition. However, a limitation of our study is the inability to distinguish between endogenous MMPs and venom-derived SVMPs. To determine whether WEL acts by direct inhibition of venom metalloproteinases (SVMPs) or indirectly by modulation of host proteases/inflammatory cascades, follow-up studies are warranted. These could include cell-free SVMP enzyme assays and kinetic analyses with purified SVMPs or venom, gelatin zymography, and binding studies (SPR/ITC or mass spectrometry) to test for direct interaction. Complementary approaches such as temporal (preincubation vs post-treatment) *in vivo* protocols, use of selective host MMP inhibitors, and proteomic profiling of venom-specific cleavage products would help discriminate a direct neutralizing action from secondary host-mediated effects. Together, these experiments clarify the molecular target(s) of WEL and better define its translational potential as an adjunctive therapy for snakebite-induced AKI.

Molecular imaging has shown that MPO regulates MMP activity.^50^ MPO activity increases in skeletal muscle following Bj envenomation,^51^ and we also observed increased renal MPO activity, potentially contributing to MMP activation. However, WEL did not reduce the MPO activity, suggesting that WEL-mediated MMP inhibition occurs through an MPO-independent pathway. The endoplasmic reticulum (ER) plays a crucial role in proteostasis, managing protein synthesis, folding, and post-translational modification. In ischemia-reperfusion-induced AKI, ER stress-mediated unfolded protein response (UPR) may trigger apoptosis.^52^ Given the disrupted kidney cytoarchitecture observed after Bj exposure, we hypothesized that nitrite accumulation could induce ER stress and apoptosis. However, activation of the UPR markers tested was not detected at the specific 24 h time point under the current experimental conditions. A broader kinetic study might be required to fully exclude this pathway. WEL’s known nephroprotective mechanisms include: (i) inhibition of NF-κB signaling in podocytes under doxorubicin-induced injury,^23^ (ii) upregulation of the antiapoptotic phosphatase PTPN2 in LPS-injured proximal tubules,^24^ and (iii) reduction in ROS, inflammatory cytokines, and cell damage.^25,28,53,54^ In a study using doxorubicin-treated MCP-5 cells, WEL inhibits IKK, preventing NF-κB translocation and decreasing inflammatory mediators such as IL-6, MCP-1, TNF-α, and TGF-β1.^27^ WEL also increased antioxidant enzyme activity (SOD, CAT, and GSH-Px) and reduced MDA and ROS levels—established oxidative stress markers. Although classical inflammatory- and oxidative-stress-related signaling pathways, such as NF-κB and MAPKs, are known contributors to kidney injury, their evaluation in the present model was limited. In our study, kidney nitrite levels were elevated after Bj envenomation, contributing to nephrotoxicity and impaired function.^55^ *Bothropoides insularis* venom induces oxidative stress in proximal tubular cells.^56^ As an antioxidant, WEL (5 mg/kg) reduced nitrite accumulation and preserved renal function.

Given the multifactorial pathogenesis of Bj venom, including systemic and local effects, our findings provide important insight into a specific therapeutic window targeting venom-induced acute kidney injury (sAKI). Although this study is limited to a preclinical model, the ability of WEL to preserve glomerular function, reduce fibrosis, and modulate inflammatory mediators suggests a potential clinical relevance. These nephroprotective effects may contribute to the development of adjunctive therapies to complement antivenom treatment, particularly in scenarios where renal involvement is prominent and antivenom alone is insufficient.

## Conclusion

This study demonstrates the nephroprotective effect of WEL (5 mg/kg) against *B. jararacussu*-induced sAKI. Envenomation resulted in both functional and structural kidney damage, including hyperfiltration, proteinuria, and impaired Na^+^ handling. WEL effectively mitigated these alterations by preserving the glomerular integrity, reducing collagen deposition, and normalizing MMP activity and nitrite levels. Although WEL has known antioxidant properties, it did not modulate MPO activity or ER stress markers, suggesting that its protective effects occur via ER stress–independent mechanisms. These findings highlight the therapeutic potential of WEL in the context of sAKI.

## Methods

### Ethics Statement

This study was conducted in accordance with the standards of good research practice and approved by the Ethics Committee on Animal Use (CEUA) at the Federal University of Rio de Janeiro (UFRJ) under protocol number 009/22.

### Bj Venom and WEL Solution

*B. jararacussu* (Bj) was obtained from the Instituto Vital Brazil (Niteroi, Brazil). A fresh venom solution was prepared by dissolving 225 mg of lyophilized venom in 40 mL of saline solution, followed by gentle mixing for 10 min at room temperature. The same venom batch was used throughout the study.

Wedelolactone (WEL) was initially dissolved at a concentration of 5 mg/mL in dimethyl sulfoxide (DMSO) and subsequently diluted with saline solution (0.9% NaCl) to achieve the desired doses (2, 5, and 10 mg/kg) for administration. At the 10 mg/kg WEL dose, the final DMSO concentration was 0.2%, which is within the range considered safe according to previous studies.^37–39^ We chose these 3 different doses based upon Puzari et al., Tu et al., and Yang et al.^57–59^ For the MMP activity and oxidative stress assays, we selected the 5 mg/kg dose because it is the most promising dosage, as it effectively prevented alterations in kidney physiological parameters, cortical and medullary collagen deposition, and kidney cytoarchitecture damage induced by Bj.^60^

### Experimental Design

Male Wistar rats (100–125g) were obtained from the “*Biotério Central de Ratos”* at UFRJ, Brazil. The rats were housed in an appropriate vivarium under controlled temperature conditions (23 ± 3 °C) and a standard 12/12 h light/dark cycle. They were provided with water and food *ad libitum* throughout the study.

The rats were randomly assigned to two primary experimental groups:

i. Ctrl group (*n* = 48): Intramuscular (IM) administration of 0.9% saline solution in the posterior region of the right thigh.
ii. Bj group (*n* = 48): IM administration of 3.5 mg/kg Bj venom in the same region, as described previously.^14^

Two hours after envenomation, both Ctrl and Bj groups were further subdivided into WEL-treated subgroups, receiving one of the three different WEL doses (IM; 2, 5, or 10 mg/kg) in the posterior region of the left thigh. This created the following final groups: Ctrl, Ctrl + W2, Ctrl + W5, Ctrl + W10, Bj, Bj + W2, Bj + W5, and Bj + W10 (*n* = 12 per group).

After WEL administration, the rats were individually housed in metabolic cages to collect 24 h of urine (UV) and 24 h of water intake (WI) measurements. At the end of the experimental period, the rats were euthanized and blood samples and the kidneys were collected.

Immediately after kidney harvesting, the left kidney was longitudinally sectioned. One-half was immersed in 10% buffered formalin for histological studies, while the remaining half and the right kidney were reserved for biochemical studies.

### Measure of Muscle Injury

Muscle injury was induced by injecting the venom into the anterior region of the right hind limb of a rat, targeting the full length of the underlying muscles. After 24 h, the affected area was identified through visual inspection, and the diameter of the injured region was measured using a caliper. Measurements were taken along the longest axis of the visible lesion to estimate the extent of muscle damage resulting from venom injection. All analyses were conducted based on this single administration.^62,63^

### Kidney Histology

Longitudinal sections of the left kidney (4 μm; 4 kidneys per group) were embedded in paraffin and stained with hematoxylin-eosin (HE) to evaluate kidney cytoarchitecture and picrosirius red to assess collagen deposition.^64,65^ Histological analyses were performed using a DP72 Microscope Digital Camera attached to an Olympus BX53F microscope (Olympus, Japan).

Glomerular morphometric analysis was conducted using histological digital images of 21 glomeruli per kidney per rat (7 from each pole and 7 from the central cortical region) at 400× magnification. The study included the total area of the renal corpuscle, the glomerular area, and Bowman’s space (the difference between the renal corpuscle and the glomerular area). These parameters were quantified using ImageJ software by manually delineating the regions within the images, calibrated against a reference scale using, according to ref 63. Collagen deposition was quantified according to the protocol described in ref 66, by measuring the percentage of stained area using ImageJ in photomicrographs (×200 magnification) from 21 nonoverlapping microscopic fields per tissue section per rat, encompassing cortical and medullary tubules.

Histological and morphometric analyses were performed by an investigator blinded to the experimental groups (HMCP, ESB, LA, and JO).

### Kidney Function Analysis

Kidney function was assessed as described in refs 14,64 . Blood samples were collected posteuthanasia in heparinized tubes and centrifuged at 3000*g* for 10 min to separate the plasma fraction for Na^+^, creatinine, and blood urea nitrogen (BUN) analysis. Urine samples were centrifuged for 5 min to remove sediments before analyzing Na^+^, creatinine, and proteinuria.

The concentration of Na^+^ in urine (U_Na_V) and blood was measured by flame spectrometry (Analyzer 910 MS, Analyzer, São Paulo, Brazil). Urinary (UCre) and plasma creatinine (PCre), BUN, and proteinuria were measured by spectrophotometry using specific colorimetric kits (Gold Analisa, Belo Horizonte, Brazil). Albuminuria was quantified by separating urinary proteins through electrophoresis, followed by analysis with ImageJ software (National Institutes of Health, Bethesda, MD). The creatinine clearance calculated the glomerular filtration rate (GFR) and is expressed as μL/min. Filtered load Na^+^ (FL_Na_; expressed in mEq/min) was determined by the product between GFR and Na^+^ concentration in the blood and the fractional excretion of Na^+^ (FE_Na_) was calculated by the percentage of Na^+^ filtered detected in the urine.

### Primary Kidney Na^+^ Transporters Activity

Kidney cortex homogenates were prepared as previously described.^67^ Total protein concentration was determined using the Lowry method,^68^ with bovine serum albumin (BSA) as standard. Samples were stored at −20 °C until analysis.

The activities of (Na^+^ + K^+^)-ATPase and Na^+^-ATPase were determined as described in ref 69. Enzymatic activity was quantified by the amount of inorganic phosphate (*P_i_*) released from ATP hydrolysis in the presence and absence of specific inhibitors: ouabain for (Na^+^+K^+^)-ATPase and furosemide for Na^+^-ATPase.

### Western Blot

Cortex homogenates (100 μg of protein) were separated by electrophoresis on polyacrylamide gel (SDS PAGE ranging from 7.5% to 12%) and transferred to a nitrocellulose membrane (10600003; GE Healthcare Life Sciences, Freiburg, Germany). After blocking with 5% milk for 1 h at room temperature, the membranes were incubated with specific primary antibody (Table 1) for 16 h at 4 °C. Following incubation, the membranes were washed using TBS-T (Tris 10 mM pH 7,4, containing 0.1% Tween-20) and then incubated with secondary fluorescent antibodies (antimouse IRDye 680RD and antirabbit IRDye 800CW, Li-Cor) diluted 10-fold compared to the primary antibodies (Table 3). Immunofluorescence signals were detected using the Odyssey Infrared Imaging System (Li-Cor Bioscience, Lincoln, NE) and band intensities were quantified using ImageJ software. The membranes were then stripped and reprobed with a monoclonal β-actin antibody, which served as a loading control.

**Table 3:** Primary Antibodies for Target Protein Detection in the Western Blot Assay

target protein	antibody source	catalog number	dilution
(Na^+^ + K^+^)-ATPase	Signia-Aldrich (Saint Louis, MO)	A276	1:1000
ATF4	Cell Signaling Technology (Danvers, MA)	D4B8	1:500
Bcl-2	Signia-Aldrich (Saint Louis, MO)	SAB4500003	1:500
Caspase 12	Signia-Aldrich (Saint Louis, MO)	PRS2327	1:1000
CHOP	Cell Signaling Technology (Danvers, MA)	L63F7	1:1000
GRP78	Santa Cruz Biotechnology (Santa Cruz, CA)	SC13968	1:200
β–actina	Signia-Aldrich (Saint Louis, MO)	A5316	1:5000

### Kidney Matrix Metalloproteinase (MMP) Activity

Kidney cortex homogenates were prepared by maceration followed by repeated freeze–thaw cycles in a lysis buffer containing 50 mM Tris-HCl, 150 mM NaCl, 5 mM CaCl_2_, 0.05%, and 1% Triton X-100, pH 7.6. The buffer was supplemented with the following protease inhibitors: 10 μM cysteine peptidase inhibitor (E–64), 5 mM serine peptidase inhibitor (PMSF), and 5 μM aspartic peptidase inhibitor (pepstatin A). Homogenates were centrifuged at 10,000*g* for 15 min at 4 °C, and the supernatants were collected for proteolytic activity assay. The protein concentration was determined using the method described in ref 61.

MMP activity was assessed using the fluorogenic substrate DNP-Pro-Leu-Gly-Met-Trp-Ser-Arg (Sigma-Aldrich, Saint Louis, MO), as described in refs 64,70 . Substrate cleavage was monitored continuously with a spectrofluorometer (SpectraMax Gemini XPS, Molecular Devices, CA) at an excitation wavelength of 280 nm and an emission wavelength of 360 nm. A 10 mM stock solution of the fluorogenic substrate was prepared in dimethyl sulfoxide (DMSO). Reactions were initiated by the addition of substrate (25 μM) to the extract (40 μg protein) in a final volume of 100 μL of the lysis buffer in the absence or presence of 5 mM 1,10-phenanthroline (PHEN), a metalloproteinase inhibitor. The reaction mixtures were incubated at 37 °C for 30 min. Self-liberation of the fluorophore was controlled over the same time interval.^65,71^

### Oxidative Stress Assays

Kidney cortex homogenate was analyzed for nitrite quantification following the Griess reaction methodology. A 2.3 mmol portion of sulfanilamide in 3% (v/v) phosphoric acid aqueous solution and a 0.2 mmol of *N*-1-naphthylethylenediamine dihydrochloride in 3% (v/v) phosphoric acid aqueous solution were prepared. The samples were mixed with these solutions in a microplate and incubated at room temperature for 30 min. Next, the OD of the solution was measured at 540 nm with a microplate reader UV/vis spectrophotometer (Molecular Devices SpectraMax 250 microplate reader, San Jose, CA). A calibration curve was plotted as different sodium nitrite concentrations against OD.^72^ Myeloperoxidase (MPO) activity in kidney tissue was measured with the HTAB, TMB, and hydrogen peroxide method. Samples were centrifuged with HTAB at 14,000*g* for 15 min. The supernatant was collected and incubated with TMB for 5 min at 37 °C. Hydrogen peroxide was added, and the mixture was incubated for 10 min at 37 °C. Then, sodium acetate buffer was added, and the OD of the solution was measured at 630 nm with a microplate reader UV/vis spectrophotometer (Molecular Devices SpectraMax 250 microplate reader, San Jose, CA, USA) as previously described.^73^

### Statistical Analyses

Data are presented as the mean ± SEM, with the number of samples (*n*) provided in the corresponding tables and figures. Multiple group comparisons were performed using one-way analysis of variance (ANOVA) followed by Sidak’s post hoc test. Statistical analysis was conducted to compare WEL treatment effects in healthy rats (Ctrl vs Ctrl + W2, Ctrl + W5 or Ctrl + W10 groups), the impact of Bj venom exposure (Ctrl vs Bj groups) and the effects of WEL treatment following Bj envenomation (Bj vs Bj + W2, Bj + W5 or Bj + W10 groups). A p value <0.05 was considered statistically significant. Statistical tests and graphs generation were performed using GraphPad Prism 8.0.2 software (GraphPad Inc., La Jolla, CA).

## Supplementary Material



## Data Availability

The data underlying this study are not publicly available due to the large volume and complexity of the raw data. The data are available from the corresponding author upon reasonable request.

## Notes

Images related to the evaluation of protein content by Western blotting presented in this manuscript: Cropped Western blotting membrane showing Na^+^/K^+^-ATPase protein content and β-actin as the corresponding loading control (Fig. S1). Full-length, uncropped Western blotting membrane showing GRP78 (Fig. S2), CHOP (Fig. S3), ATF4 (Fig. S4), Bcl-2 (Fig. S5), and Caspase-12 (Fig. S6) protein contents, along with their respective beta-actin loading controls (PDF)

## Abbreviations

sAKI: snakebite-induced acute kidney injury
Wel: wedelolactone
Bj: *Bothrops jararacussu*
IgG: immunoglobulin G
FE_Na_: fractional sodium excretion
EP: *Eclipta prostate*
Ctrl: control
WI: water intake
UV: urinary volume
GFR: glomerular filtration rate
PCre: plasma creatinine concentration
BUN: blood urea nitrogen
FL_Na_: sodium filtered load
U_Na_V: urinary sodium excretion
HE: hematoxylin-eosin
MMP: matrix metalloproteinase
ER stress: endoplasmic reticulum stress
MPO: myeloperoxidade
CKD: chronic kidney disease
RAS: renin-angiotensin system
ROS: reactive oxidative stress
UPR: unfolding protein response
SVMPs: snakebite metalloproteinases
SVSPs: snakebite serine proteases
LAO: l-amino acid oxidase
