# Supplementary material for: Nephroprotective Effects of Wedelolactone against Snake Venom-Induced Acute Kidney Injury: Insights into Experimental Envenomation
Source: ACS Omega. 2026 Mar 4;11(10):15956–69. doi: 10.1021/acsomega.5c09974 (PMC13000654; doi:10.1021/acsomega.5c09974)
Supplement: Supplementary file 1 [file ao5c09974_si_001.pdf]

Nephroprotective effects of wedelolactone against snake venom-induced acute kidney injury: insights into experimental envenomation

Mayara A. Romanelli[1,2], Pâmella D. Nogueira-Souza[1], Dayene S. Gomes[1,2], Gabriel A. Bastos[1,2], Helen M.C. Pinto[3], Ellen S. Brito[3], Lucas Albernaz[3], Tamires Pereira[1,2], Janaína Oliveira[3], Simone S.C. Oliveira[4], Carolinne S. Amorim[1], André L.S. Santos[4], João A. Moraes[1], Sabrina R. Gonsalez[3], Paulo A. Melo[3*], Lucienne S. Lara[1,2,*]

[1] Instituto de Ciências Biomédicas, Universidade Federal do Rio de Janeiro, Rio de Janeiro, Brazil, 21941-590.

[2]Centro de Pesquisa em Medicina de Precisão, Universidade Federal do Rio de Janeiro, Rio de Janeiro, Brazil, 21941-902.

[3] Instituto de Ciências Médicas, Universidade Federal do Rio de Janeiro, Macaé, Rio de Janeiro, Brazil, 27930-560.

[4]Instituto de Microbiologia Paulo de Góes, Universidade Federal do Rio de Janeiro, Rio de Janeiro, Brazil, 21941-901.

Supporting information

Figure S1. Cropped Western blotting membrane showing Na^+/K^+-ATPase protein content and β-actin as the corresponding loading control.

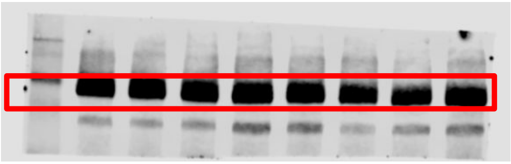

NKA	110 KDa	Na^+/K^+-ATPase 1:1000 SIGMA

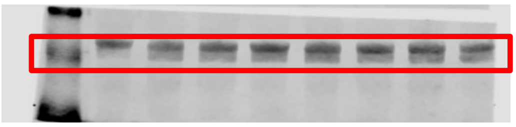

β-actin	42 KDa	1:1000

Figure S2. Full-length, uncropped Western blotting membrane showing GRP78 protein content and β-actin as the corresponding loading control.

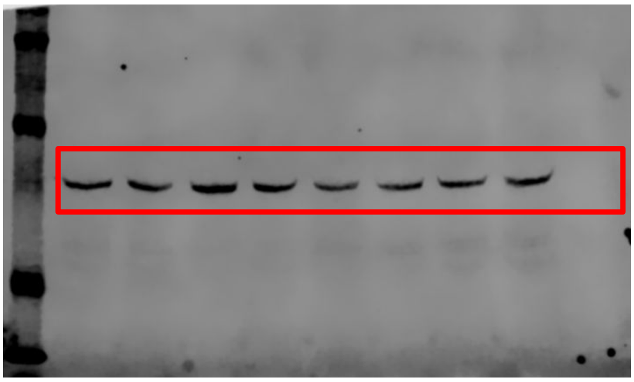

GRP78	78 KDa	GRP78 (SC13968) Anti-mouse Santa Cruz

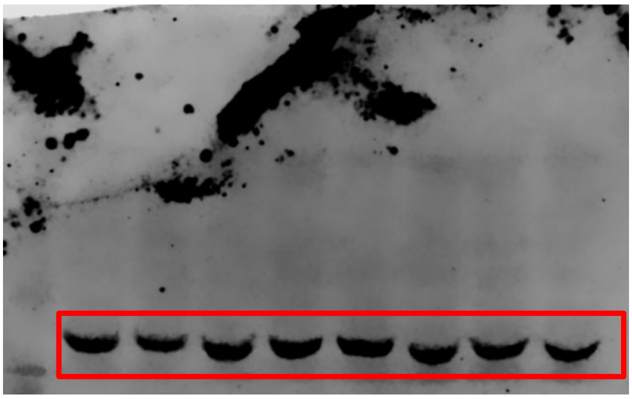

β-actin	42 KDa	1:1000

Figure S3. Full-length, uncropped Western blotting membrane showing CHOP protein content and β-actin as the corresponding loading control.

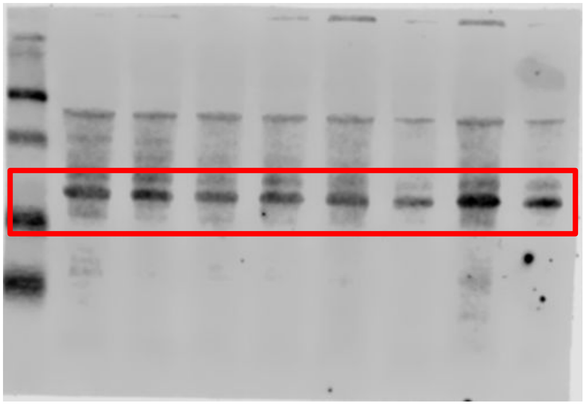

CHOP	27 KDa	CHOP 1:1000 (L63F7) Anti-Mouse CellSignaling

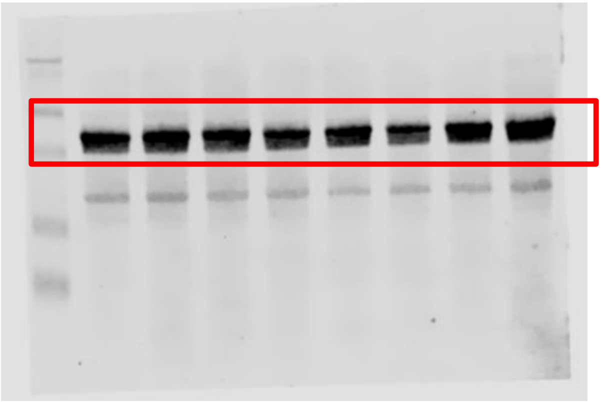

β-actin	42 KDa	1:1000

Figure S4. Full-length, uncropped Western blotting membrane showing ATF-4 protein content and β-actin as the corresponding loading control.

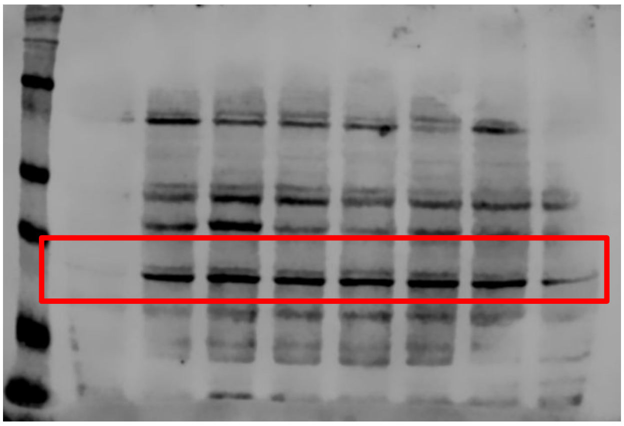

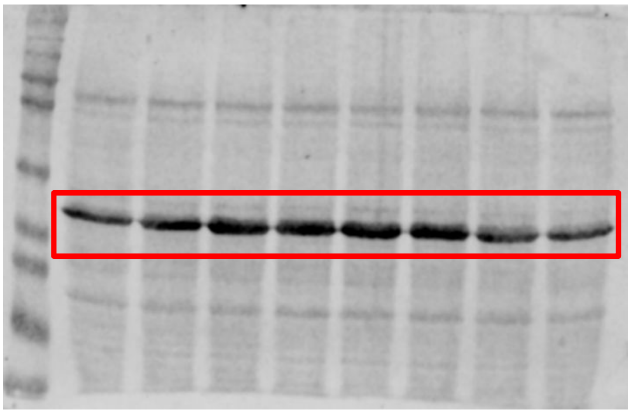

Figure S5. Full-length, uncropped Western blotting membrane showing Bcl-2 protein content and β-actin as the corresponding loading control.

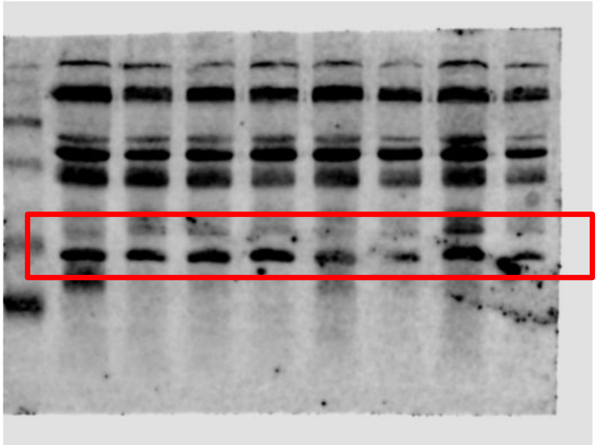

Bcl-2	26 KDa	BCL-2 1:500 (SAB4500003) Anti-Rabbit Lt:210774 SIGMA

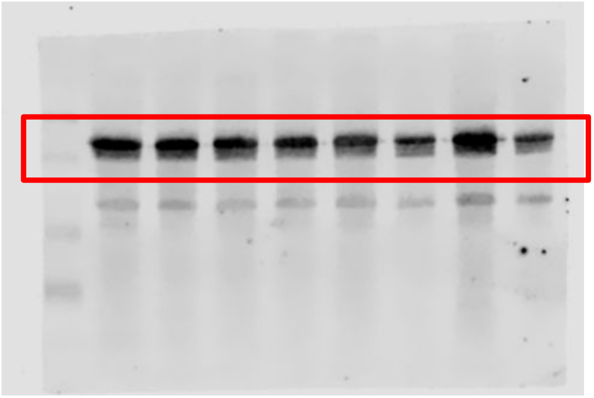

β-actin	42 KDa	1:1000

Figure S6. Full-length, uncropped Western blotting membrane showing Caspase-12 protein content and β-actin as the corresponding loading control.

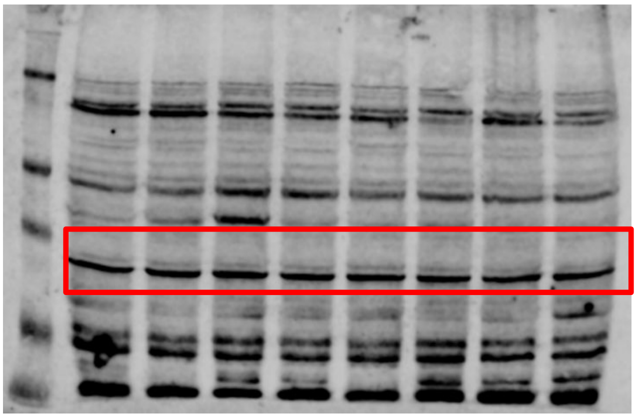

Caspase-12	51KDa	Caspase 12 1:1000 (PRS2327) Anti-Rabbit Lt.2327104 SIGMA

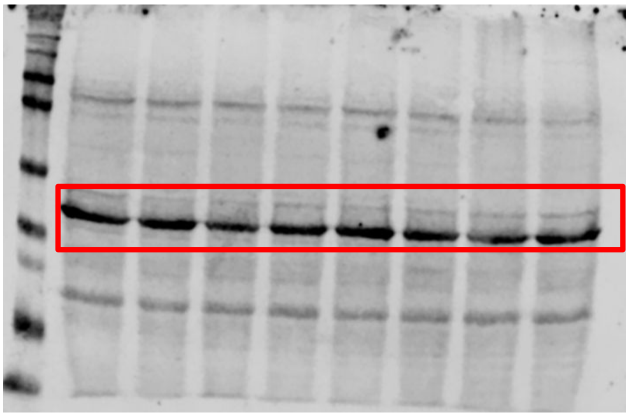

β-actin	42KDa	1:1000
